# MitraClip^®^ procedure as bridge to left ventricular assist device: Enable extracorporeal membrane oxygenation weaning and reduce perioperative risk

**DOI:** 10.1177/02676591211065251

**Published:** 2022-01-03

**Authors:** Martin O. Schmiady, Stephan Winnik, Dominique Bettex, Raed Aser

**Affiliations:** 1 Division of Cardiac Surgery, University Heart Center, 27243 University Hospital Zurich, Zurich, Switzerland; 2 Department Cardiology, University Heart Center, 27243 University Hospital Zurich, Zurich, Switzerland; 3 Institute of Anaesthesiology, University of Zurich and University Hospital Zurich, Zurich, Switzerland

**Keywords:** MitraClip^®^, extracorporeal membrane oxygenation, left ventricular assist device, Impella^®^ microaxial blood pump, mitral regurgitation, end-stage heart failure, cardiac shock

## Abstract

Secondary mitral valve regurgitation is a frequent consequence of left ventricular dysfunction in patients with severe heart failure. The management of this disease can be challenging since it often culminates in refractory pulmonary edema and multi-organ failure. We present the case of a 50-year-old male who was admitted in cardiogenic shock following myocardial infarction. After successful revascularization, percutaneous mitral valve repair using the MitraClip^®^ device enabled weaning from extracorporeal membrane oxygenation followed by the implantation of a left ventricular assist device as bridge to transplant.

## Case

**Figure 1.**
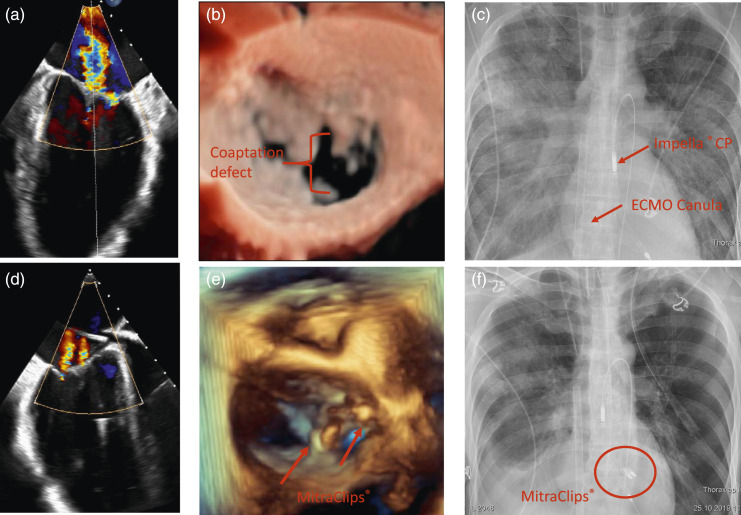
Transesophageal echocardiography of the mitral valve with severe mitral regurgitation due to coaptation defect (0.7 mm) between P3 and A3 (A and B). TEE image and 3D image after clip implantation showing a significant reduction of the regurgitation jet (D and E). Regression of pulmonary edema after MitraClip^®^ implantation, with two clips in place (F), compared to preoperative conditions (C).

A 50-year-old male patient presented with acute ST-elevation infarction. He underwent a left heart catheter, revealing a total occlusion of the proximal circumflex artery. Under intra-aortic balloon pump support, he underwent interventional recanalization with coronary drug-eluting stent implantation. A transthoracic echocardiographic examination revealed a severe secondary mitral valve regurgitation (grade III) due to left ventricular dilatation (LVEDD = 56 mm) and a severely reduced of the left ventricular ejection fraction (LVEF 10%) (Figure 1(a) and (b)). New York Heart Association (NYHA) classification was grade four. In the further course, the patient deteriorated and mechanical circulatory support using a veno-arterial extracorporeal membrane oxygenation (VA-ECMO) became necessary. However, left ventricular ejection fraction remained minimal, pulse pressure deteriorated and the left ventricle became progressively dilated leading to a grade IV secondary mitral regurgitation. Therefore, the intra-aortic balloon pump was replaced by an Impella^®^ CP pump (ECMELLA) to unload the left ventricle. Several attempts to wean from mechanical circulatory support (MCS) using an extended catecholamine regime with dobutamine and norepinephrine failed because of recurrent pulmonary edema and the onset of multiple organ failure. In this critical situation, an LVAD implantation would have been associated with a prohibitively high mortality risk. Decision was made for percutaneous MitraClip^®^ implantation to allow ECMELLA weaning and organ recovery.

After clipping (two clips), mitral valve regurgitation was significantly reduced to grade I (Fig. D and E). There was no significant stenosis after clipping (dp mean = 3 mmHg). LVEDD dropped from 56 to 50 mm. After MitraClip^®^ implantation, the patient stabilized and pulmonary edema was resolved (Figures 1(c) and (f)). Within 11 days, the Impella^®^ pump as well as the ECMO could be successfully weaned and explanted. After organ recovery, the patient underwent elective LVAD implantation. Postoperative course was uneventful, and patient was discharged to rehabilitation 1 month later in good clinical condition, and is currently at home awaiting transplantation.

## Discussion

Percutaneous mitral valve repair (PMVR) using the MitraClip^®^ system is a rapidly developing approach for selected patients suffering from mitral valve regurgitation. Clip implantation can be performed safely in high-risk heart failure patients with functional mitral regurgitation, whose surgical risk is prohibitively high or who are considered inoperable.^1^ PMVR has been shown to improve hemodynamic parameters, symptoms, and re-hospitalization in this challenging patient group.^2^ Although reverse cardiac remodeling at mid-term follow-up has been reported after successful MitraClip^®^ implantation in heart failure patients with depressed left ventricular ejection fraction, left ventricular dysfunction seems to progress in patient with end-stage heart failure.^3^ Even if the progression of the underlying disease cannot be stopped, MitraClip^®^ implantation can temporarily improve hemodynamics and reduce symptoms. This enables high-risk patients in multi-organ failure to recover and to reduce the risk of subsequent surgery. In our case, this strategy enabled successful ECMELLA weaning and organ recovery, allowing for the implantation of a continuous-flow left ventricular assist device (CF-LVAD). LVAD implantation was performed in the usual way, without any interaction with the implanted two clips. Based on the available literature, implantation of CF-LVAD appears safe in patients with previously placed MitraClips^®^, with no need for additional open mitral valve surgery.^4^ This case may serve as proof-of-concept and demonstrates the potential role of PMVR in stabilizing patients, and supporting organ recovery to allow optimal conditions for elective surgery, and potentially reducing perioperative risk and mortality. Additional treatment costs for the MitraClip device must be taken into account, but in our case, could be amortized by shortening the intensive care period.

## Conclusion

The presented case shows that PMVR using the MitraClip^®^ system is feasible under simultaneous VA-ECMO and Impella^®^ support and can be effective in stabilization, recompensation, and recovery in severely compromised patients with severe secondary mitral valve regurgitation and cardiogenic shock. This concept provides a reasonable bailout strategy in selected patients allowing for their stabilization before LVAD implantation, likely to reduce perioperative mortality and to improve the outcome.
